# Association between components of the delirium syndrome and outcomes in hospitalised adults: a systematic review and meta-analysis

**DOI:** 10.1186/s12877-021-02095-z

**Published:** 2021-03-05

**Authors:** Zoë Tieges, Terence Quinn, Lorn MacKenzie, Daniel Davis, Graciela Muniz-Terrera, Alasdair M. J. MacLullich, Susan D. Shenkin

**Affiliations:** 1 Geriatric Medicine, Edinburgh Delirium Research Group, Usher Institute, University of Edinburgh, Edinburgh, Scotland UK; 2 School of Health and Life Sciences, Glasgow Caledonian University, Glasgow, Scotland UK; 3 Institute of Cardiovascular and Medical Sciences, University of Glasgow, Glasgow, UK; 4 Academic and Clinical Central Office for Research and Development, University of Edinburgh, Edinburgh, UK; 5 MRC Unit for Lifelong Health and Ageing at University College London, London, UK; 6 Centre for Clinical Brain Sciences and Dementia Prevention, University of Edinburgh, Edinburgh, UK

**Keywords:** Delirium, Mortality, Altered mental status, Arousal, Attention, Systematic review

## Abstract

**Background:**

Delirium is a heterogeneous syndrome with inattention as the core feature. There is considerable variation in the presence and degree of other symptom domains such as altered arousal, psychotic features and global cognitive dysfunction. Delirium is independently associated with increased mortality, but it is unclear whether individual symptom domains of delirium have prognostic importance. We conducted a systematic review and meta-analysis of studies in hospitalised adults in general settings to identify the relationship between symptom domains of delirium and outcomes.

(PROSPERO: CRD42018093935).

**Methods:**

We searched MEDLINE, EMBASE, PsycINFO, CINAHL, clinicaltrials.gov and the Cochrane Central Register of Controlled Trials from inception to November 2019. We included studies of hospitalised adults that reported associations between symptom domains of delirium and 30-day mortality (primary outcome), and other outcomes including mortality at other time points, length of stay, and dementia. Reviewer pairs independently screened articles, extracted data, and assessed risk of bias (Risk of Bias Assessment tool for Non-randomized Studies) and quality of evidence using the Grading of Recommendations, Assessment, Development and Evaluation framework. We performed random-effects meta-analyses stratified by delirium domain where possible.

**Results:**

From 7092 citations we included 6 studies (6002 patients, 1112 with delirium). Higher mortality (ranging from in-hospital to follow-up beyond 12 months) was associated with altered arousal (pooled Odds Ratio (OR) 2.80, 95% Confidence Interval (CI) 2.33–3.37; moderate-quality evidence), inattention (pooled OR 2.57, 95% CI 1.74–3.80; low-quality evidence), and in single studies with disorientation, memory deficits and disorganised thoughts. Risk of bias varied across studies but was moderate-to-high overall, mainly due to selection bias, lack of blinding of assessments and unclear risk of selective outcome reporting. We found no studies on the association between psychotic features, visuospatial deficits or affective disturbances in delirium and outcomes, or studies reporting non-mortality outcomes.

**Conclusions:**

Few studies have related symptom domains of delirium to outcomes, but the available evidence suggests that altered arousal and inattention in delirium are associated with higher mortality than normal arousal and attention in people with or without delirium. Measurable symptom domains of delirium may have value in predicting survival and stratifying patients for treatment. We recommend that future delirium studies report outcomes by symptom domain.

**Supplementary Information:**

The online version contains supplementary material available at 10.1186/s12877-021-02095-z.

## Background

Delirium is an acute, serious neuropsychiatric syndrome of cognitive, psychiatric and motor abnormalities which is commonly triggered by acute illness, surgery, trauma or medications. Delirium affects at least 1 in 6 hospitalised older patients [1, 2]. It is often highly distressing for patients and their carers [3, 4]. Most cases of delirium resolve within days, though around 20% persists for weeks or months [5].

The core feature of delirium is inattention of acute onset. However, the neuropsychological and neuropsychiatric features of delirium are wide ranging. The syndrome of delirium may also include disturbances in level of arousal, memory, orientation (i.e. awareness to time, place and person), visuospatial ability, psychotic features (i.e. misperceptions, hallucinations and illusions), psychomotor activity, thought process (disorganised thinking) and affect to varying degrees [6, 7].

Delirium in hospitalised adults is independently associated with multiple adverse outcomes including a two-fold increased risk of mortality (Hazard Ratio (HR) 1.95, 95% Confidence Interval (CI) 1.51–2.52 [8]), longer stay in hospital, new institutionalisation (odds ratio (OR), 2.41, 95% CI 1.77–3.29 [8]) and an 8-fold increased risk of dementia (OR 8.7, 95% CI 2.1–35 [9]) [1, 2, 9]. Delirium severity scores, reflecting the summed ordinal measures of various symptom domains of the delirium syndrome [10–13], have also been shown to predict outcomes including mortality [14], though the evidence is mixed [15]. However, the construct of delirium severity is complex and lacks a clear operational definition, with several domains such as altered level of arousal, psychosis, and inattention present to varying degrees in tools for assessing severity. This heterogeneity makes the study of the relationships between delirium severity and prognosis challenging.

It is unclear whether the individual symptom domains of the delirium syndrome are associated with adverse patient outcomes, and whether specific delirium symptoms drive the observed association with these outcomes. In most existing delirium severity scales, the individual symptom domains included in the scale are equally weighted (e.g. [11, 13].

Some studies have examined subtypes (hypo- and hyperactive, and mixed delirium) in relation to outcomes, mostly suggesting that hypoactive presentations of delirium are indicative of a worse prognosis [16–18]. However these studies did not consider the individual symptom domains. Altered arousal has been found to be predictive of mortality - a recent systematic review in adult hospitalised patients found an adjusted 6-fold increase in risk of 30-day mortality in patients with altered arousal [19], but none of the studies considered delirium. Of interest, subsyndromal delirium, in which patients exhibit one or more symptoms but do not meet full criteria for delirium, has been shown to carry intermediate risk [20].

A better understanding of the associations between individual symptom domains of delirium and patient outcomes is needed to inform patient risk stratification and prioritisation of interventions for preventing and treating delirium. In other words, identifying predictors of poor outcomes in delirium would assist clinicians to risk stratify patients in order to focus management and guide discussions on prognosis. Furthermore, advancing our understanding of the individual domains of delirium that contribute to poor outcomes could lead to a more refined operationalisation of delirium itself. This is important because the key features of delirium lack explicit and agreed definitions, and there is continued debate about operationalisation and assessment methods of these features [6, 21]).

## Objectives

We conducted a systematic review to establish if the presence of individual symptom domains of delirium in hospitalised adults, in studies which included patients with delirium, is associated with increased mortality and other adverse patient outcomes. The potential effect on outcomes of individual symptom domains of delirium was studied in both patients with and without a delirium diagnosis.

## Methods

This systematic review was reported in accordance with the Preferred Reporting of Items in Systematic Reviews and Meta-Analysis (PRISMA) statement [22].

### Protocol and registration

The study protocol was prospectively registered with PROSPERO, the international prospective register of systematic reviews (http://www.crd.york.ac.uk/PROSPERO/ registration number CRD42018093935).

### Selection criteria

Study inclusion criteria were: (1) hospitalised patients aged 18 and over; (2) patients in whom an assessment of delirium was made using standardised diagnostic criteria or validated tools (e.g. Confusion Assessment Method (CAM) and its derivatives including the CAM for the Intensive Care Unit, Delirium Rating Scale-Revised 98 (DRS-R98), Memorial Delirium Assessment Scale, Delirium Index, Delirium Observation Screening Scale, Delirium Symptom Overview, or 4AT); (3) collected data on at least one of our pre-specified outcomes (defined below); and (4) studies which reported the outcome stratified by presence or absence of individual symptom domains typically considered to be part of the delirium syndrome, including disturbance in attention, level of arousal, memory, orientation, language, visuospatial ability, affect and thought process (disorganised thinking), and psychotic features (including misinterpretations, hallucinations and illusions).

Exclusion criteria were studies in: (1) patients with delirium tremens; (2) the intensive care unit or high dependency unit or a setting focused on providing specialist end of life care (e.g. hospice); and (3) mixed settings unless data from patients admitted to hospital could be separated.

The primary outcome was mortality at 30 days. Secondary outcomes were mortality at other timepoints including in-hospital mortality, as well as length of hospital stay (days), readmission, new admission to care home, duration of delirium, incident dementia and quality of life (after a delirium episode) as measured by a validated questionnaire.

All study designs were considered.

### Data sources

An inclusive search strategy was developed with an experienced librarian using selected keywords relating to delirium, key delirium symptom domains of interest and outcome. The search terms used to identify delirium were based on the validated delirium search syntax produced by the National Institute for Health and Clinical Excellence (NICE) clinical guidance for delirium (Additional file 1: Databases search strategies) [23]. The following databases were searched: MEDLINE® (OVID), EMBASE (OVID), PsycINFO (EBSCO), CINAHL (EBSCO), clinicaltrials.gov and the Cochrane Central Register of Controlled trials from inception to 9 May 2018, with an updated search until 21 November 2019.

No restrictions on language or publication date were imposed. We conducted forward citation searches of included articles and checked reference lists of included articles for further articles of potential relevance. Where data were not presented as outcome data stratified by delirium domain, we contacted the authors to request either individual patient-level data or summary data.

Professionals from the European Delirium Association, American Delirium Society and Australasian Delirium Association were contacted by email and social media to identify other relevant published or unpublished articles or data for inclusion. We also contacted relevant experts through social media.

### Study selection and data extraction

Pairs of review authors (LM and ZT; TQ and ZT) independently reviewed all titles and abstracts for eligibility. They then independently evaluated full texts for inclusion, resolving any disagreement by discussion or, if required, by another review author (SDS). Data were extracted independently by each reviewer and comprised study design and setting, population (e.g. reason for admission), co-morbid illness or illness severity (e.g. dementia or cognitive impairment, depression, frailty), sex, age range, total number of patients and total number with delirium and/or dementia, severity of delirium, method for diagnosing delirium, time of patient assessment, mortality, length of hospital stay, readmission to hospital, new admission to care home, quality of life assessment, statistic used, adjustments made to the analysis, conclusion of the study and any study quality measures. We primarily sought hazard ratios (HR) or odds ratios (OR) of mortality. Where there was ambiguity over results, the authors were contacted for clarification.

### Risk of bias assessment and grading of quality of evidence

The risk of bias was assessed with a modified version of The Risk of Bias Assessment tool for Non-randomized Studies (RoBANS) [24], with pre-determined criteria for low or high risk for each domain (Additional file 2: RoBANS quality assessment criteria). The reviewer pairs independently assessed risk of bias and agreed by consensus.

An assessment of overall quality of evidence was made according to the Grading of Recommendations, Assessment, Development and Evaluation (GRADE) framework [25]. We assessed risk of bias, consistency of results (heterogeneity), directness (applicability of included studies to research question), precision (based on CIs of summary estimate), and publication bias. Because we anticipated that most studies would be observational in nature, we did not downgrade the quality of the evidence for this factor.

### Synthesis of Results

Statistical heterogeneity was assessed using a visual assessment of forest plots supplemented with a quantitative assessment (Higgins I^2^ [26]). Studies were synthesized using meta-analyses where reporting was sufficiently homogeneous (agreed by consensus). Quantitative analysis was performed using Comprehensive Meta-Analysis (CMA) Version 3 software [27]. To allow summary analysis, HRs were converted to ORs using CMA software. We calculated summary estimates where data were reported on the same predictor variable from three or more studies. Where mortality was reported at multiple time points, the last point of follow-up was used in the analysis. Since we only identified one study on the primary outcome, 30-day mortality, we synthesised the data across all mortality time points (ranging from in-hospital mortality to follow-up beyond 12 months); this was a deviation from the pre-specified study protocol. We conducted stratified analyses by delirium symptom domain using random-effects models to calculate pooled ORs and 95% CIs, supplemented with a narrative analysis.

A sensitivity analysis was performed excluding one study that included only patients with delirium [28].

## Results

### Study selection

**Fig. 1:**
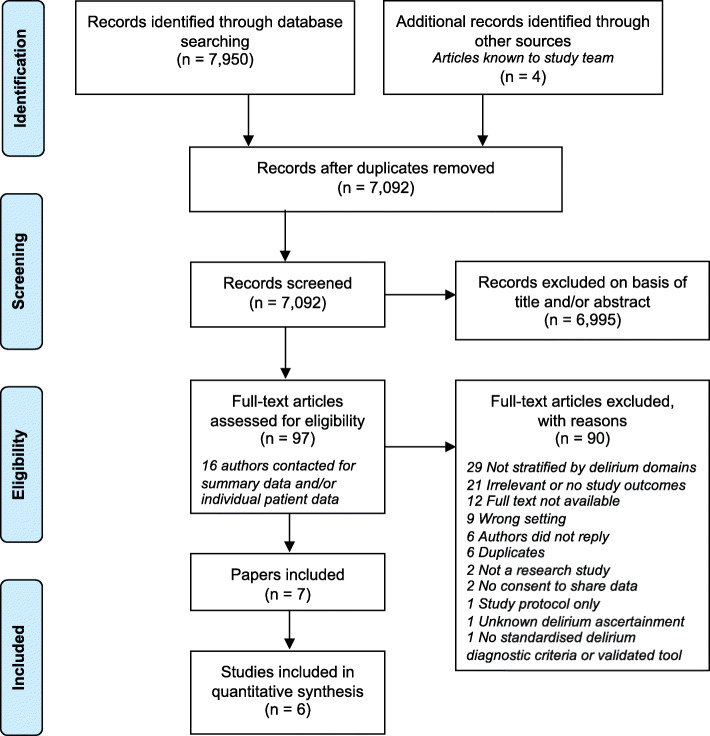
PRISMA flow chart of study selection [22]

We identified 7950 articles from our initial search, and 7092 after initial deduplication (Fig. 1). Following title and abstract screening, 97 records had full-text review and seven articles were included reporting six different studies [28–33]. We contacted 16 authors (including from these six studies) to request summary data, of which 10 authors replied: 4 authors shared data, 4 authors confirmed they did not report outcomes of interest stratified by delirium symptom domains, 1 study did not provide summary data due to a conflict of interest (i.e. the authors plan on publishing these data at a later date) and 1 author was unable to share data due to lack of patient consent. The main reason for exclusion of articles was that results stratified by delirium symptom domains were not reported and not provided by authors. Two articles reported results from the same cohort [29, 34]: the article which provided additional prognostic information on a sub-domain of delirium was included in the synthesis of results [29].

### Study characteristics

All six studies were cohort studies, four prospective and two retrospective. Studies were published between 2013 and 2020 from the UK, USA, Italy, and Brazil (Table 1). Three studies were identified through conference abstracts; the authors of one study provided summary data [32] and authors of the other two studies shared the unpublished manuscript [28] or the accepted manuscript [33]. All studies recruited hospitalised patients from acute medical wards, orthopaedic wards or emergency departments. The sample size varied from 108 to 2521, with a total of 6002 patients included in the review population of which 1112 (18.5%) had delirium as assessed by diagnostic criteria or a validated diagnostic instrument. Patients in one study all had a delirium diagnosis [28]. The remaining five studies reported a prevalence of delirium ranging from 2.9 to 44.4% (this wide range reflecting different selection criteria). Mean or median age ranged from 77 to 84.4 years. Baseline co-morbidity data were collected in all studies and included dementia diagnosis (4 studies, prevalence 38.9 to 57%), cognitive impairment (1 study) and illness severity (3 studies).

**Table 1:** Descriptive characteristics from included studies

Study ID	Country	Study Design	Setting	Total N	Delirium N (%)	Dementia N (%)	Age (y), mean (SD)	Female (%)	Inclusion Criteria	Exclusion Criteria	Co-morbid illness / illness severity	Delirium Diagnosis/Screening Tool	Delirium diagnosed by
Bellelli 2015^a^	Italy	Prospective cohort study	Acute hospital medical wards	2521	72 (2.9)	28 (38.9)	79.1 (7.3)	50.8	Aged ≥65; SBT assessment ≤72 h of admission	Comatose; Incomplete data	Dementia: ICD-9;Cognitive Impairment: SBT; Co-morbid illness/Illness Severity: Not reported	ICD-9-CM	Attending Physician
Diwell 2018	UK	Prospective cohort study	Acute hospital medical wards	610	271 (44.4)	129 (47.6)	83 (7)	59	Aged ≥70;Unplanned medical admission; admitted ≥48 h	Non-English speaker	Dementia/Cognitive Impairment: Not reported; Co-morbid illness: CCI; Illness Severity: APACHE II	s-CAM	Psychiatrist
Hall 2013^a^	UK	Prospective cohort study	Hospital orthopaedic wards	108	44 (40.7)	Not reported	80	61	Aged ≥60; Admitted to orthopaedic wards; Acute hip fracture & spinal anaesthesia	Nursing home resident; Taken oral/inhaled steroids ≤10 weeks; Parkinson’s disease /comorbid disease with prognosis ≤1 year; Communication difficulties	Dementia/Cognitive impairment: Not reported; Co-morbid illness: CCI; Illness Severity: APACHE II	CAM; DRS-R98; EDTB; OSLA; RASS	Geriatrician
Han 2017	USA	Secondary analysis of two prospective cohort studies	Emergency Department	1084	155 (14.3)	91 (8.4)	77 (71–84) median with IQR	59.4	Aged ≥65; In ED ≤ 12 h	Non-English speaker; Persistent coma; Unable to follow simple commands before acute illness	Dementia/Cognitive impairment: IQCODE; Co-morbid illness/Illness Severity: Not reported	CAM-ICU; RASS	Research Assistants; Emergency Medical Technicians; Paramedics; Psychiatrist
Jackson 2018^b^	UK	Prospective cohort study	Acute hospital medical wards	125	125 (100)	71 (57)	84.4 (6.5)	63	Aged ≥70 with unplanned medical admission; Meet DSM-IV-TR criteria	Unable to communicate due to sensory impairment; Inability to communicate in English; End of life	Dementia/ Cognitive impairment: IQCODE; Co-morbid illness: CCI; Illness Severity: APACHE II	CAM; DSM-IV-TR; DRS-R98; OSLA	Geriatrician
Garcez 2020	Brazil	Retrospective cohort study	Geriatric hospital wards	1554	445 (28)	872 (56)	81 (8)	61	Aged ≥65 nonelective hospitalisation; arousal assessment on admission using validated instrument	Discharged or died within 38 h of admission	Dementia/Cognitive impairment: IQCODE and Clinical Dementia Rating; Co-morbid illness: CCI; Illness Severity: Not reported	s-CAM	Clinical geriatrics fellow and revised by geriatrician

*Notes:* Data are given as means (standard deviation, SD), median (interquartile ranges, IQR) or number (%). SBT: Short Blessed Test; CCI: Charlson Comorbidity Index, a weighted scale used to measure comorbidity burden; APS/APACHE II: Acute Physiology Score/Acute Physiology and Chronic Health Evaluation, both scales used to quantify severity of illness; IQCODE: Informant Questionnaire on Cognitive Decline in the Elderly; RASS: Richmond Agitation-Sedation Scale; s-CAM: short Confusion Assessment Method; CAM-ICU: CAM for the Intensive Care Unit. OSLA: Observational Scale of Level of Arousal (score range 0–15), a bidirectional bedside scale of arousal. EDTB: Edinburgh Delirium Test Box. DRS-R98: Delirium Rating Scale-Revised 98; ICD; International Classification of Diseases; DSM: Diagnostic and Statistical Manual of Mental Disorders, ED: Emergency Department. ^a^Author provided additional summary data. ^b^Author provided draft unpublished article

Five studies used a delirium screening tool to assess delirium: the CAM [35] or its variants (short CAM, or s-CAM, and CAM for the Intensive Care Unit) and/or the DRS-R98 [11]. Two studies reported diagnosing delirium according to the Diagnostic Statistical Manual of Mental Disorders, 4th Edition Text Revision (DSM-IV-TR) [28] or the International Classification of Mental and Behavioural Disorders 9th Revision Clinical Modification (ICD-9-CM) [31] (Table 1).

### Neuropsychological domains

*Arousal*: Five studies assessed level of arousal with various tools: s-CAM [31, 33]; Glasgow Coma Scale [33]; Richmond Agitation-Sedation Scale (RASS; 29, 32); and/or the Observational Scale for Level of Arousal (OSLA; 28, 32). Studies which used a scale for assessing arousal dichotomised the scale to determine whether altered arousal was present or absent; the degree (or severity) of arousal disturbance was not considered. Diwell et al. [31] did not define ‘altered level of consciousness’, but it was clear from the paper that this term referred to altered level of arousal as assessed with the short CAM [36]. One study distinguished between reduced and increased arousal [29], another study used GCS with scores ≤13 to indicate decreased arousal [33], and the remaining studies did not specify whether arousal was increased or decreased.

*Attention*: Attention was assessed in three studies, using either a computerised test of focused and sustained attention (Edinburgh Delirium Test Box [37]) [32], counting backwards from 20 to 1 and the Months of the Year backwards test [30], or patient observation (as part of the s-CAM) [31]. As with altered arousal, inattention was coded in the studies as present or absent, and the degree of inattention was not considered in the analyses.

*Other neuropsychological domains*: in single studies, the presence of *disorientation* (name current year, month and time of day) and *memory deficits* (immediate and delayed recall), both part of the Short Blessed Test [38], and *disorganised thinking* (included in the CAM assessment [35]) was assessed. There were no studies that collected data on the association between psychotic features, visuospatial deficits or affective disturbances in delirium with mortality, or secondary non-mortality outcomes.

Three studies reported on the timing of the initial assessment: day 1 [32] or day 3 [29, 31] after admission or at time of enrolment [29]. No other studies reported on timing and frequency of assessments.

### Outcome measures

One study reported the primary outcome of 30-day mortality [33]. Three studies reported mortality at other follow-up timepoints, namely at 4, 6 and/or 12 months [28, 29, 32]. One study reported in-hospital mortality [30] and another study reported mortality according to the UK Office of National Statistics where death was flagged by a certified death certificate, however details of the exact timepoint of this were not provided [31] (Table 1).

We found no studies which reported on the association between delirium symptom domains and any of the non-mortality secondary outcomes.

All studies reported unadjusted and adjusted data. Multivariable Cox proportional hazard models were performed to produce HRs of survival in four studies [28, 29, 31, 33], and a form of logistic regression with ORs was used in two studies [30, 32]. All studies reported 95% CIs. One study reported model adjustments for prevalent delirium [33]. No other studies adjusted their models for delirium or other domain impairments.

### Risk of bias

**Fig. 2:**
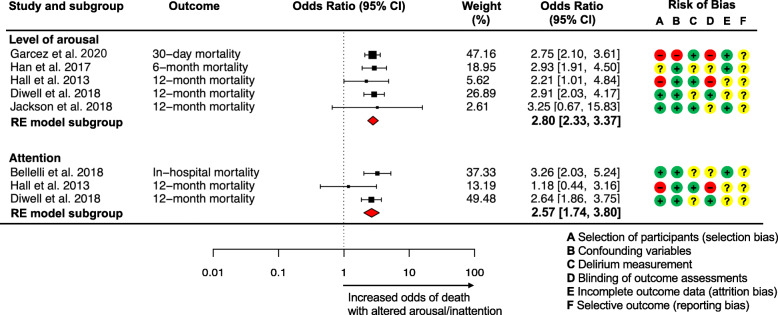
Forest plot of the association between altered level of arousal and inattention with mortality, and risk of bias summary graph of all included studies (low (−), high (+) or unclear (?))

Risk of bias varied across studies but was moderate to high overall (Fig. 2). This was mainly due to: possible selection bias, either because studies recruited patients from a convenience sample and/or because of the retrospective study design used [29, 32, 33]; lack of reported blinding of outcome assessments [28–30, 32, 33]; and the absence of a pre-published protocol to allow assessment of selective outcome reporting (all studies). The risk of bias was mostly low for the consideration of confounding variables, with sufficient information in individual studies regarding baseline variables such as dementia, co-morbidities, functional dependence, frailty, and illness severity, and inclusion of these variables in multivariate analyses. Most studies used a validated scale to assess delirium, although training and frequency of assessments were often poorly reported, resulting in unclear risk of bias [29–31]. One study [31] diagnosed delirium by retrieving ICD-9 codes from web-based case reports without describing the method in detail (unclear risk of bias). Four studies [28–30, 33] addressed how missing data were treated and accounted for in the analysis (low risk of bias), whereas the remaining two studies [31, 32] did not report the number and reasons for loss to follow-up (unclear risk of bias).

### Synthesis of results

#### Altered level of arousal

Altered (versus normal) level of arousal (with or without delirium) was associated with higher mortality at 30 days, and at 4, 6, and 12 months [28, 29, 32, 33] (Table 1 and Additional file 3: Table 2 expanded). Random effects meta-analysis indicated that presence of altered arousal (compared to normal arousal) was associated with higher mortality across follow-up time-points ranging from 30 days to 12 months (pooled OR 2.80, 95% CI 2.33–3.37, I^2^ = 0%, *N* = 3481 (1040 delirium), risk of bias moderate-to-high: possible selection bias, unclear or lack of blinding of assessments and unclear risk of selective outcome reporting) (Fig. 2). The quality of evidence for the association between altered arousal and higher mortality was moderate, downgraded because of high risk of bias (all but one study had unclear or high risk of bias ratings in at least three RoBANS domains), indirectness, and publication bias.

**Table 2:** Association between delirium symptom domain and mortality in included studies

Delirium symptom domain	Study	Mortality	Ratio type	Comparison	Assessment tool used for symptom domain	Ratio (95% CI)	***p***-value	Adjustments
Altered level of arousal								
	Garcez 2020	30-day mortality	OR	Altered vs. Normal arousal	GCS	1.62 (1.13–2.33)	0.009	Age, sex, delirium, comorbidities, nutritional status, baseline cognitive impairment, polypharmacy
	Han 2017	6-month mortality	HR	Decreased arousal/delirium vs. No delirium	RASS	1.4 (0.9–2.1)	> 0.05	Age, sex, comorbidity burden, severity of illness, dementia, functional dependence, admission status
	Hall 2018	12-month mortality	OR	Altered vs Normal arousal	OSLA	2.21 (1.01–4.86)	< 0.05	Age, sex, comorbidity burden, severity of illness
	Diwell 2018	Mortality at any timepoint^a^	HR	Altered arousal/Delirium vs. No delirium	s-CAM	1.33 (0.98–1.79)	0.063	Age, sex, co-morbidity burden, pressure sores, severity of illness
	Jackson 2018	12-month mortality	HR	Decreased vs. Normal arousal/delirium	OSLA	1.09 (1.02–1.18)	0.01	Age
Inattention								
	Bellelli 2015	In-hospital mortality	OR	No delirium	SBT	3.26 (2.03–5.24)^b^	< 0.0001	Age, sex, nursing home residence, prior hospitalisation (6-month period), co-morbidity, dementia
	Hall 2018	12-month mortality	OR	Altered vs. Normal arousal	EDTB	1.18 (0.44–3.16)	> 0.05	Age, sex, comorbidity burden, severity of illness
	Diwell 2018	Mortality at any timepoint^a^	HR	No delirium	s-CAM	1.24 (0.92–1.67)	0.152	Age, sex, co-morbidity burden, pressure sores, severity of illness
Disorientation								
	Bellelli 2015	In-hospital mortality	OR	No delirium	SBT	3.85 (2.43–6.10)^b^	< 0.0001	Age, sex, nursing home residence, prior hospitalisation (6-month period), co-morbidity, dementia
Memory deficits								
	Bellelli 2015	In-hospital mortality	OR	No delirium	SBT	2.92 (1.33–6.39)^b^	0.005	Age, sex, nursing home residence, prior hospitalisation (6-month period), co-morbidity, dementia
Disorganised thought								
	Diwell 2018	Mortality at any timepoint^a^	HR	No delirium	s-CAM	1.42 (1.05–1.92)	0.024	Age, sex, co-morbidity burden, pressure sores, severity of illness
Psychotic features (hallucinations and delusions)								
	No studies available	⁃	⁃	⁃	⁃	⁃	⁃	⁃
Visuospatial deficits								
	No studies available	⁃	⁃	⁃	⁃	⁃	⁃	⁃
Affective disturbances								
	No studies available	⁃	⁃	⁃	⁃	⁃	⁃	⁃

*Notes:* HR = Hazard Ratio; OR: Odds Ratio; CI: Confidence Interval; GCS: Glasgow Coma Scale; RASS: Richmond Agitation-Sedation Scale; OSLA: Observational Scale of Level of Arousal; s-CAM: short Confusion Assessment Method; SBT: Short Blessed Test; EDTB: Edinburgh Delirium Test Box. ^a^ Death was flagged by the UK Office of National Statistics and certified by a death certificate. ^b^ OR statistics were obtained from authors

A sensitivity analysis excluding the Jackson et al. study [28] did not alter these findings (pooled OR 2.80; 95% CI 2.32–3.37).

Garcez et al. [33] was the only study reporting on the association between level of arousal and 30-day mortality, the primary outcome. In this retrospective study, altered (compared to normal) arousal was independently associated with higher 30-day mortality after adjusting for confounders including delirium, both when arousal was defined using s-CAM criteria (HR 2.33, 95% CI 1.66–3.27) and GCS scores (HR 1.62, 95% CI 1.13–2.33; GCS ≤ 13). The adjustment for delirium may have led to over-adjustment especially when using s-CAM criteria to define both arousal and delirium status.

Hall et al. [32] reported that altered level of arousal was associated with increased risk of death at 12 months (OSLA: HR 2.21, 95% CI 1.01–4.86; RASS: HR 2.13, 95% CI 1.03–4.4).

Han et al. [29] compared the associations between each arousal subtype in delirium (normal/increased/decreased, using non-delirious patients as the reference group) and 6-month mortality in patients presenting in the emergency department. Of all three delirium arousal subtypes, delirium with normal arousal had the highest 6-month mortality (HR 3.1, 95% CI 1.3–7.4) although the sample size for this subgroup was very small (*N* = 15). Furthermore, this was the only paper that found an association between *increased* arousal in delirium and mortality (HR 1.4, 95% CI 0.9–2.1; *N* = 8), again using a very small sample size. The direction of the effect was similar to that found for *decreased* arousal in delirium (HR 1.4, 95% CI 0.9–2.1; *N* = 132). A different paper describing the same cohort further reported that patients with altered arousal, irrespective of delirium status, were more likely to die within 6 months (HR 1.73, 95% CI 1.21–2.49) compared to those with normal arousal [34].

Jackson et al. [28] only included patients with delirium on admission who were followed up at 4 and 12 months. Delirium with reduced arousal and hypoactive motor symptoms in hospital was associated with higher mortality at 12 months when compared to delirium patients with hyperactive features (i.e. mixed and hyperactive motor subtypes; HR 1.09, 95% CI 1.02–1.18). In addition, analysis of arousal subtypes, informed by scores on the OSLA, DRS-R98 and clinical descriptions, found an association between the hypoactive (vs. hyperactive/mixed) subtype with higher mortality at 4 months (HR 3.18, 95% CI 1.13–8.93).

Diwell et al. [31] did not find a significant association between level of consciousness (i.e. altered arousal) and mortality in adjusted analysis (HR 1.33 95% CI 0.98–1.79).

#### Inattention

Results from the meta-analysis showed that presence of inattention (pooled across patients with and without delirium) was associated with higher mortality compared to normal attention and/or no delirium (pooled OR 2.57, 95% CI 1.74–3.80; I^2^ = 0%, risk of bias moderate: possible selection bias, unclear or lack of blinding of assessments, and unclear risk of selective outcome reporting; 3239 patients (387 delirium)). The quality of evidence for the association between inattention and higher mortality was low, downgraded because of risk of bias (all three studies had an unclear or high risk of bias in at least three RoBANS domains), imprecision, indirectness, and publication bias.

Only one of three original studies assessing attention reported a statistically significant association between inattention and higher mortality. Bellelli et al. [30] reported an association between inattention in older patients (with delirium) and in-hospital mortality (OR 3.26, 95% CI 2.03–5.24) compared to a non-delirious group. It is noted that this study reported a lower prevalence rate of delirium (2.9%) than expected given the setting, suggesting that a proportion of cases with delirium were missed. Diwell et al. [31] reported a statistically significant association between inattention with higher mortality, but this association disappeared after adjustment for confounders (HR 1.24, 95% CI 0.92–1.67).

#### Other neuropsychological deficits

The evidence regarding other symptom domains of delirium and mortality is limited (two studies: 3131 patients, 343 with delirium [30, 31]). Bellelli et al. [30] found higher in-hospital mortality in delirium patients who had evidence of disorientation (OR 3.85, 95% CI 2.43–6.1) and memory deficits (OR 2.92, 95% CI 1.33–6.39) compared to non-delirious patients. Interestingly, the diagnosis of delirium itself was not a predictor of mortality in this study. The authors further noted that delirious patients mostly had multiple neurocognitive deficits and that patterns of inattention with memory and/or orientation deficits were shown to be more strongly associated with in-hospital mortality compared to having only one neurocognitive deficit.

Diwell et al. [31] found an association between the presence of disorganised thinking in patients with delirium (compared to no delirium) and higher mortality (timescale not specified but identified through the UK Office for National Statistics; HR 1.42, 95% CI 1.05–1.92).

## Discussion

### Summary of evidence

This systematic review demonstrates that the evidence base regarding the association between symptom domains of delirium and mortality and other outcomes is small and has marked heterogeneity. A meta-analysis of five studies found that *altered level of arousal* was associated with 2.8-fold higher mortality, ranging from 30-day mortality to follow-up beyond 12 months after hospital admission, although the quality of evidence was low to moderate. Meta-analysis of three studies found that *inattention* associated with delirium conferred a 2.6-fold higher risk of mortality ranging from in-hospital mortality to follow-up beyond 12 months.

The evidence on the association between other delirium domains and mortality was sparse, with a lack of data on psychotic features, visuospatial deficits and affective disturbances.

We found no studies reporting on other outcomes than mortality.

### Strengths and limitations

This was a comprehensive systematic review evaluating over 7000 references using an inclusive search strategy with no initial restrictions on language, study design or date. All references, abstracts and full texts were reviewed by independent assessors. We contacted delirium researchers around the world to identify unpublished studies. We followed up conference abstracts and performed forward citation searches. We contacted authors for data and clarification. In light of predicted significant heterogeneity, a random effects model was used in the meta-analysis.

Several limitations must be acknowledged. Studies included in the meta-analysis were heterogeneous, with variation in sample size, delirium prevalence and setting. The number of studies (*N* = 6) was too small to permit additional analyses (e.g., stratified analyses by setting or delirium subgroups). The study with the largest sample size (*N* = 2521) reported a delirium prevalence of only 2.9% (*N* = 72), which suggests that some patients with delirium may have been misclassified as non-delirious. This could in turn have led to a decrease in the size of the association seen with outcome. Although we searched conference proceedings and contacted delirium research organisations, we may have missed valuable contributions in the grey literature. Also, there were no full texts available for 12 relevant articles; these were mainly conference abstracts and authors did not respond to our request for the full paper or study data. We could not identify full text articles of these records on a further check in November 2020.

Whilst we used a comprehensive list of search terms in an effort to capture the wide variation of descriptions of the symptom domains of delirium and assessment methods for measuring these symptom domains, we cannot rule out the possibility that we may have missed some studies.

Studies differed with regards to the comparison group used: these were either patients without delirium, patients (either with or without delirium) who did not display the relevant symptom domain, or patients with delirium only who did not display the symptom domain. We did not carry out subgroup analyses because insufficient data on subgroups were provided, and it was therefore not possible to distinguish the impact of the individual delirium symptom domains versus the overall impact of a delirium diagnosis.

The included studies were of mixed quality overall, and heterogeneous in the populations studied (with delirium prevalence ranging between 2.9 to 100% due to selection methodology, and dementia prevalence ranging between 38.9 to 56%), methods for assessing arousal and attention, methods of delirium assessment, adjustment for dementia and illness severity, and the length of follow-up of mortality. Given this clinical and methodological heterogeneity, the exploratory findings from the meta-analysis should be interpreted with caution. Studies mostly did not specify the timing and frequency of delirium assessments, and it was also unclear if some patients developed persistent delirium which could have affected the relationship between delirium symptom domains and outcomes. Nevertheless, the positive association between altered level of arousal in delirium and higher mortality was reasonably consistent across studies. Studies also adjusted for key confounders including age, comorbidity burden and dementia status.

### Interpretation and implications for clinical practice and future research

The current evidence base regarding the association between delirium symptom domains and mortality is small and inconclusive. Nonetheless, the present findings suggest that individual symptom domains of delirium may have prognostic value. This appears to be especially true for altered level of arousal. One study reported that altered arousal in itself was a stronger indicator of mortality than a diagnosis of delirium [32], suggesting that assessment of arousal in delirium may have prognostic value over and above diagnostic classification. Interestingly, a recent systematic review found that altered arousal in the absence of delirium is a strong prognostic marker of mortality [19]. In contrast to a delirium diagnosis which can be difficult to ascertain particularly in the absence of a reliable informant history, level of arousal can be quickly and objectively assessed at the bedside with an observational tool such as the RASS [39], OSLA [40], or by using delirium scales with embedded level of arousal measurement such as the 4AT [41]. Level of arousal is also an important component of immediate clinical assessment e.g. using the GCS, or the AVPU scale (A: alert; V: responds to voice; P: responds to pain; U: unresponsive) [42], which has been integrated into clinical ‘early warning scores’ and is therefore part of regular observations in hospital. Further, the degree (or severity) of disturbances in arousal and attention might add additional prognostic information beyond a binary score (i.e., present/absent), but this requires further study.

More broadly, delirium assessment tools, including tools for assessing delirium severity and monitoring for recovery from delirium, may be challenging to administer in busy clinical environments, and knowledge of the symptom domains that drive the association with adverse outcomes could be translated into more tailored delirium assessment tools.

Altered level of arousal, inattention and other symptom domains of delirium are commonly assessed as part of delirium assessment tools in research and clinical practice (e.g. 3D-CAM [43], DRS-R98 [11] etc.), but individual test item or subscale scores are rarely reported. Also, there remains considerable uncertainty with respect to the conceptualisation and assessment of the component features of delirium. We recommend more explicit and consistent reporting of delirium symptom domain scores and the assessment tools used (for example Neerland et al. [44]). Such an approach of increased transparency and consistency of reporting would allow for improved assessment of study quality and facilitate further research into the prognostic utility of the individual symptom domains of delirium.

Future research is needed to examine the prognostic importance of delirium symptom domains not only in relation to mortality, but also other adverse outcomes known to be linked to delirium (including persistent cognitive impairment and dementia, and anxiety, depressive, and post-traumatic stress disorder symptoms [9, 45]). Such knowledge could inform development of interventions targeted at delirium patients with deficits in specific symptom domains, and also inform stratification of patients for treatment and/or intervention trials. This would require prospective cohort studies evaluating sufficient numbers of unselected patients, and using standardised methods for assessing the individual symptom domains of delirium. Better characterisation of presence and severity of pre-existing cognitive impairment prior to admission could be achieved using a retrospective informant questionnaire such as the Informant Questionnaire of Cognitive Decline in the Elderly (IQCODE [46]) and/or an informant-based dementia severity rating instrument. Further, standardisation of methods for assessing and reporting symptom domains of the delirium syndrome is also needed (both the presence and severity of symptoms), because this will increase transparency and comparability of studies and facilitate data sharing efforts and meta-analyses of individual patient data [47]; efforts to harmonize and calibrate the symptom domain measures are also needed to enable effective comparison and pooled analyses of study results. With regards to outcome measures, there is a clear need for consensus on time points for follow-up to allow comparison between studies. Finally, this review highlights the need for evidence-based development and refinement of tools for measuring delirium severity, a construct which is widely used yet imprecisely defined and variably assessed [48].

Delirium is a syndrome with a wide spectrum of clinical presentations. The present findings suggest that specific phenotypes of delirium (altered arousal, inattention) in older hospitalised adults are associated with worse outcomes and should be a focus of future research, but the impact of impairments in other symptom domains has not been evaluated. Exploration of the factors behind the heterogeneity of delirium presentations and outcomes may improve patient outcomes through risk stratification and proactive management and inform future research into delirium pathophysiology and treatment.

## Conclusions

This systematic review and meta-analysis showed that few published studies have assessed the relationship between symptom domains of delirium and mortality. The findings suggest that specific measurable symptom domains of the delirium syndrome, especially altered arousal, may have value in predicting survival. If confirmed in future studies, this would mean that patients with delirium of the altered-arousal subtype should be identified as having a higher risk of death. This review provides the foundation for future work to help advance our understanding of the delirium syndrome and to clarify risk factors and outcomes in order to focus management and guide discussions on prognosis, and facilitate the development of targeted interventions for delirium.

## Supplementary Information

**Additional file 1.** Databases search strategies.

**Additional file 2.** Risk of Bias Assessment tool for Non-randomized Studies (RoBANS) quality assessment criteria.

**Additional file 3.** Association between delirium symptom domain and mortality (Table 2 expanded).

## Data Availability

The datasets used and/or analysed during the current study are available from the corresponding author on reasonable request.

## Abbreviations

4AT: 4 ‘A’s Test
APACHE II: Acute Physiology and Chronic Health Evaluation
AVPU: Alert, responds to Verbal stimulus, responds to Painful stimulus and Unresponsive
CAM: Confusion Assessment Method
s-CAM: short CAM
CAM-ICU: CAM for the Intensive Care Unit
CI: Confidence Interval
DRS-R98: Delirium Rating Scale-Revised 98
DSM-IV-TR: Diagnostic Statistical Manual of Mental Disorders, 4th Edition Text Revision
ICD-9-CM: International Classification of Mental and Behavioural Disorders 9th Revision Clinical Modification
GCS: Glasgow Coma Scale
GRADE: Grading of Recommendations, Assessment, Development and Evaluation
HR: Hazard Ratio
IQCODE: Informant Questionnaire on Cognitive Decline in the Elderly
NICE: National Institute for Health and Clinical Excellence
OR: Odds Ratio
OSLA: Observational Scale of Level of Arousal
PRISMA: Preferred Reporting Items for Systematic Reviews and Meta-Analyses
RASS: Richmond Agitation-Sedation Scale
RoBANS: The Risk of Bias Assessment tool for Non-randomized Studies
s-CAM: short Confusion Assessment Method
