# Correction to: Association between components of the delirium syndrome and outcomes in hospitalised adults: a systematic review and meta-analysis

**DOI:** 10.1186/s12877-021-02419-z

**Published:** 2021-09-09

**Authors:** Zoë Tieges, Terence Quinn, Lorn MacKenzie, Daniel Davis, Graciela Muniz-Terrera, Alasdair M. J. MacLullich, Susan D. Shenkin

**Affiliations:** 1 Geriatric Medicine, Edinburgh Delirium Research Group, Usher Institute, University of Edinburgh, Edinburgh, Scotland, UK; 2 School of Health and Life Sciences, Glasgow Caledonian University, Glasgow, Scotland, UK; 3 Institute of Cardiovascular and Medical Sciences, University of Glasgow, Glasgow, UK; 4 Academic and Clinical Central Office for Research and Development, University of Edinburgh, Edinburgh, UK; 5 MRC Unit for Lifelong Health and Ageing at University College London, London, UK; 6 Centre for Clinical Brain Sciences and Dementia Prevention, University of Edinburgh, Edinburgh, UK

**Correction to: BMC Geriatr 21, 162 (2021)**

**https://doi.org/10.1186/s12877-021-02095-z**

Following publication of the original article [1], the authors reported that funding of one of the authors had accidentally been omitted.

The original article [1] has been updated.
