# Supplementary material for: Association between components of the delirium syndrome and outcomes in hospitalised adults: a systematic review and meta-analysis
Source: BMC Geriatr. 2021 Mar 5;21:162. doi: 10.1186/s12877-021-02095-z (PMC7934253; doi:10.1186/s12877-021-02095-z)
Supplement: Supplementary file 1 — Additional file 1. Databases search strategies. [file 12877_2021_2095_MOESM1_ESM.docx]

**Association between components of the delirium syndrome and outcomes in hospitalised adults: a systematic review and meta-analysis.**

Authors:

Zoë Tieges^1,2^ zoe.tieges@ed.ac.uk

Terence Quinn^3^ Terry.Quinn@glasgow.ac.uk

Lorn MacKenzie^4^ lorn.mackenzie@nhslothian.scot.nhs.uk

Daniel Davis^5^ daniel.davis@ucl.ac.uk

Graciela Muniz-Terrera^6^ G.Muniz@ed.ac.uk

Alasdair M. J. MacLullich^1^ a.maclullich@ed.ac.uk

Susan D. Shenkin^1^ Susan.Shenkin@ed.ac.uk

^1^Geriatric Medicine, Edinburgh Delirium Research Group, Usher Institute, University of Edinburgh, Edinburgh, Scotland, UK. ^2^School of Health and Life Sciences, Glasgow Caledonian University, Glasgow, Scotland, UK. ^3^Institute of Cardiovascular and Medical Sciences, University of Glasgow, Glasgow, UK. ^4^Academic and Clinical Central Office for Research & Development, University of Edinburgh, Edinburgh, UK. ^5^MRC Unit for Lifelong Health and Ageing at University College London, London, UK. ^6^Centre for Clinical Brain Sciences and Dementia Prevention, University of Edinburgh, Edinburgh, UK.

**Additional file 1. Database Searches**

OVID MEDLINE

1. deliri$.ti,ab.

2. (acute adj2 (confusion$ or "brain syndrome" or "brain failure" or "psycho-organic syndrome" or "organic psychosyndrome")).mp.

3. (terminal$ adj restless$).mp.

4. toxic confus$.mp.

5. delirium/

6. confusion/

7. or/1-6

8. attention.mp.

9. inattention.mp.

10. exp Arousal/

11. exp Psychotic Disorders/

12. Mental Processes/

13. HALLUCINATIONS/

14. Neurobehavioral Manifestations/

15. Behavioral Symptoms/

16. or/8-15

17. 7 and 16

OVID EMBASE

1. deliri$.ti,ab.

2. (acute adj2 (confusion$ or "brain syndrome" or "brain failure" or "psycho-organic syndrome" or "organic psychosyndrome")).mp.

3. (terminal$ adj restless$).mp.

4. toxic confus$.mp.

5. delirium/

6. confusion/ or acute confusion/

7. or/1-6

8. attention.mp.

9. inattention.mp.

10. arousal/

11. exp psychosis/

12. mental function/

13. hallucination/

14. behavior disorder/

15. or/8-14

16. 7 and 15

17. Validat$.mp. or Predict$.ti. or Rule$.mp. or (Predict$ and (Outcome$ or Risk$ or Model$)).mp. or ((History or Variable$ or Criteria or Scor$ or Characteristic$ or Finding$ or Factor$) and (Predict$ or Model$ or Decision$ or Identif$ or Prognos$)).mp. or (Decision$.mp. and ((Model$ or Clinical$).mp. or Statistical Models/)) or (Prognostic and (History or Variable$ or Criteria or Scor$ or Characteristic$ or Finding$ or Factor$ or Model$)).mp.

18. (Stratification or Discrimination or Discriminate or c-statistic or c statistic or Area under the curve or AUC or Calibration or Indices or Algorithm or Multivariable).mp.

19. or/17-18

20. 16 and 19

OVID PsycINFO

1. deliri$.ti,ab.

2. (acute adj2 (confusion$ or "brain syndrome" or "brain failure" or "psycho-organic syndrome" or "organic psychosyndrome")).mp.

3. (terminal$ adj restless$).mp.

4. toxic confus$.mp.

5. DELIRIUM/

6. MENTAL CONFUSION/

7. or/1-6

8. attention.mp.

9. inattention.mp.

10. exp physiological arousal/

11. HALLUCINATIONS/

12. cognitive impairment/

13. behavior problems

14. or/8-13

15. 7 and 14

CINAHL

1. Deliri*

2. Terminal restlessness

3. MH Delirium

4. MH Confusion

5. 1 OR 2 OR 3 OR 4

6. Inattention

7. MH Arousal

8. MH Mental Processes

9. MH Hallucinations

10. MH Neurobehavioral Manifestations

11. MH Behavioral Symptoms

12. 7 OR 8 OR 9 OR 10 OR 11

13. 5 AND 12
