# Supplementary material for: Association between components of the delirium syndrome and outcomes in hospitalised adults: a systematic review and meta-analysis
Source: BMC Geriatr. 2021 Mar 5;21:162. doi: 10.1186/s12877-021-02095-z (PMC7934253; doi:10.1186/s12877-021-02095-z)
Supplement: Supplementary file 2 — Additional file 2. Risk of Bias Assessment tool for Non-randomized Studies (RoBANS) quality assessment criteria. [file 12877_2021_2095_MOESM2_ESM.docx]

**Association between components of the delirium syndrome and outcomes in hospitalised adults: a systematic review and meta-analysis.**

Authors:

Zoë Tieges^1,2^ zoe.tieges@ed.ac.uk

Terence Quinn^3^ Terry.Quinn@glasgow.ac.uk

Lorn MacKenzie^4^ lorn.mackenzie@nhslothian.scot.nhs.uk

Daniel Davis^5^ daniel.davis@ucl.ac.uk

Graciela Muniz-Terrera^6^ G.Muniz@ed.ac.uk

Alasdair M. J. MacLullich^1^ a.maclullich@ed.ac.uk

Susan D. Shenkin^1^ Susan.Shenkin@ed.ac.uk

^1^Geriatric Medicine, Edinburgh Delirium Research Group, Usher Institute, University of Edinburgh, Edinburgh, Scotland, UK. ^2^School of Health and Life Sciences, Glasgow Caledonian University, Glasgow, Scotland, UK. ^3^Institute of Cardiovascular and Medical Sciences, University of Glasgow, Glasgow, UK. ^4^Academic and Clinical Central Office for Research & Development, University of Edinburgh, Edinburgh, UK. ^5^MRC Unit for Lifelong Health and Ageing at University College London, London, UK. ^6^Centre for Clinical Brain Sciences and Dementia Prevention, University of Edinburgh, Edinburgh, UK.

### **Additional file 2. RoBANS Quality Assessment Criteria**

| **Risk of Bias Item** | **Grading** | **Rationale/Examples** |
| --- | --- | --- |
| **Selection of participants** *(selection bias)* | Low | Sampling frame clearly described with reasonable inclusion/exclusion criteria provided, ideally allowing for inclusion of all participants admitted to the hospital (or specialist service) in whom an assessment of delirium is made using a standardised diagnostic criteria or validated tools. Exclusion of those with delirium tremens or those admitted to the intensive care unit (ICU)/high dependency unit (HDU) or setting that is focused on providing specialist end of life care. |
|  | High | Exclusions based on availability of data (e.g. admission to ICU/HDU; transfer to nursing/residential care/setting that is focused on providing specialist end of life care), exclusion of those lost to follow-up. |
|  | Unclear | Sampling frame unclear, criteria for inclusion/exclusion not provided or explained. |
| **Confounding variables** | Low | Multivariate model accounting for likely possible confounding variables. |
|  | High | No consideration of confounding variables; univariate analyses only. |
|  | Unclear | Methods for analysis not clearly described or reported. |
| **Measurement of exposure** *(performance bias)* | Low | Clearly described method on how data were collected and extracted. Best practice includes description of who performed data extraction (who performed delirium assessment), case definitions/descriptions of eligible conditions. |
|  | High | Missing data on key delirium assessment methods. |
|  | Unclear | Methods for assessing delirium not clearly described. |
| **The blinding of the outcome assessment** | Low | Blinding of outcomes detailed. |
|  | High | Blinding of outcomes not taken into consideration. |
|  | Unclear | Not reported in articles. |
| **Incomplete outcome data**  *(attrition bias)* | Low | Outcomes assessed for all included participants. |
|  | High | Missing outcome assessments. |
|  | Unclear | Outcome assessment reported as percentages without absolute values being presented, preventing assessment of completeness of outcome reporting. |
| **Selective outcome reporting**  *(reporting bias)* | Low | Reporting as per published protocol. |
|  | High | Evidence that reporting deviates from publicly accessible protocol/ |
|  | Unclear | No protocol publicly available. |

RoBANS, Risk of Bias Assessment Tool for Non-Randomised Studies.
