# Supplementary material for: Association between components of the delirium syndrome and outcomes in hospitalised adults: a systematic review and meta-analysis
Source: BMC Geriatr. 2021 Mar 5;21:162. doi: 10.1186/s12877-021-02095-z (PMC7934253; doi:10.1186/s12877-021-02095-z)
Supplement: Supplementary file 3 — Additional file 3. Association between delirium symptom domain and mortality (Table 2 expanded). [file 12877_2021_2095_MOESM3_ESM.docx]

**Association between components of the delirium syndrome and outcomes in hospitalised adults: a systematic review and meta-analysis.**

Authors:

Zoë Tieges^1,2^ zoe.tieges@ed.ac.uk

Terence Quinn^3^ Terry.Quinn@glasgow.ac.uk

Lorn MacKenzie^4^ lorn.mackenzie@nhslothian.scot.nhs.uk

Daniel Davis^5^ daniel.davis@ucl.ac.uk

Graciela Muniz-Terrera^6^ G.Muniz@ed.ac.uk

Alasdair M. J. MacLullich^1^ a.maclullich@ed.ac.uk

Susan D. Shenkin^1^ Susan.Shenkin@ed.ac.uk

^1^Geriatric Medicine, Edinburgh Delirium Research Group, Usher Institute, University of Edinburgh, Edinburgh, Scotland, UK. ^2^School of Health and Life Sciences, Glasgow Caledonian University, Glasgow, Scotland, UK. ^3^Institute of Cardiovascular and Medical Sciences, University of Glasgow, Glasgow, UK. ^4^Academic and Clinical Central Office for Research & Development, University of Edinburgh, Edinburgh, UK. ^5^MRC Unit for Lifelong Health and Ageing at University College London, London, UK. ^6^Centre for Clinical Brain Sciences and Dementia Prevention, University of Edinburgh, Edinburgh, UK

**Additional file 3.** Association between delirium symptom domain and mortality (Table 2 expanded).

| **Delirium symptom domain** | **Study** | **Mortality** | **Ratio type** | **Comparison** | **Assessment tool used for symptom domain** | **Ratio (95% CI)** | **p-value** | **Adjustments** |
| --- | --- | --- | --- | --- | --- | --- | --- | --- |
| Altered level of arousal | | | | | | | | |
|  | Garcez 2020 | 30-day mortality | OR | Altered arousal vs. Normal arousal | GCS/  s-CAM | 1.62 (1.13-2.33)/  2.33 (1.66-3.27) | 0.009 | Age, sex, delirium, comorbidities, nutritional status, baseline cognitive impairment, polypharmacy |
|  | Han 2017 | 6-month mortality | HR | Normal arousal/Delirium vs. No delirium | RASS | 3.1 (1.3-7.4) | <0.05 | Age, sex, comorbidity burden, severity of illness, dementia, functional dependence, admission status |
|  |  |  |  | Decreased arousal/delirium vs. No delirium | RASS | 1.4 (0.9-2.1) | >0.05 | Age, sex, comorbidity burden, severity of illness, dementia, functional dependence, admission status |
|  |  |  |  | Increased arousal/Delirium vs. No delirium | RASS | 1.3 (0.3-5.4) | >0.05 | Age, sex, comorbidity burden, severity of illness, dementia, functional dependence, admission status |
|  | Han 2014^c^ | 6-month mortality | HR | Altered vs Normal arousal | RASS | 1.73 (1.21-2.49) | <0.05 | Age, sex, comorbidity burden, severity of illness, dementia, functional dependence, admission status, psychoactive medications |
|  | Hall 2018 | 12-month mortality | OR | Altered vs Normal arousal | OSLA/  RASS | 2.21 (1.01-4.86)/  2.13(1.03-4.4) | <0.05 | Age, sex, comorbidity burden, severity of illness |
|  | Diwell 2018 | Mortality at any timepoint ^a^ | HR | Altered arousal/Delirium vs. No delirium | s-CAM | 1.33 (0.98-1.79) | 0.063 | Age, sex, co-morbidity burden, pressure sores, severity of illness |
|  | Jackson 2018 | 4-month mortality | HR | Decreased vs. Normal arousal /delirium | OSLA | 3.18 (1.13-8.93) | 0.03 | Age, severity of illness, frailty, comorbidity burden |
|  |  | 12-month mortality | HR | Decreased vs. Normal arousal/delirium | OSLA | 1.09 (1.02-1.18) | 0.01 | Age |
| Inattention | | | | | | | | |
|  | Bellelli 2015 | In-hospital mortality | OR | No delirium | SBT | 3.26 (2.03-5.24)^b^ | <0.0001 | Age, sex, nursing home residence, prior hospitalisation (6-month period), co-morbidity, dementia |
|  | Hall 2018 | 12-month mortality | OR | Altered arousal vs. Normal arousal | EDTB | 1.18 (0.44-3.16) | >0.05 | Age, sex, comorbidity burden, severity of illness |
|  | Diwell 2018 | Mortality at any timepoint ^a^ | HR | No delirium | s-CAM | 1.24 (0.92-1.67) | 0.152 | Age, sex, co-morbidity burden, pressure sores, severity of illness |
| Disorientation | | | | | | | | |
|  | Bellelli 2015 | In-hospital mortality | OR | No delirium | SBT | 3.85 (2.43-6.10)^b^ | <0.0001 | Age, sex, nursing home residence, prior hospitalisation (6-month period), co-morbidity, dementia |
| Memory deficits | | | | | | | | |
|  | Bellelli 2015 | In-hospital mortality | OR | No delirium | SBT | 2.92 (1.33-6.39)^b^ | 0.005 | Age, sex, nursing home residence, prior hospitalisation (6-month period), co-morbidity, dementia |
| Disorganised thought | | | | | | | | |
|  | Diwell 2018 | Mortality at any timepoint ^a^ | HR | No delirium | s-CAM | 1.42 (1.05-1.92) | 0.024 | Age, sex, co-morbidity burden, pressure sores, severity of illness |
| Psychotic features (hallucinations and delusions) | | | | | | | | |
|  | No studies available | ⁃ | ⁃ | ⁃ | ⁃ | ⁃ | ⁃ | ⁃ |
| Visuospatial deficits | | | | | | | | |
|  | No studies available | ⁃ | ⁃ | ⁃ | ⁃ | ⁃ | ⁃ | ⁃ |
| Affective disturbances | | | | | | | | |
|  | No studies available | ⁃ | ⁃ | ⁃ | ⁃ | ⁃ | ⁃ | ⁃ |

*Notes:* HR = hazard ratio; OR = odds ratio; CI = confidence interval; GCS: Glasgow Coma Scale; RASS: Richmond Agitation-Sedation Scale; OSLA: Observational Scale of Level of Arousal; s-CAM: short Confusion Assessment Method; SBT: Short Blessed Test; EDTB: Edinburgh Delirium Test Box. ^a^ Death was flagged by the UK Office of National Statistics and certified by a death certificate. ^b^ OR statistics were obtained from authors. ^c^ Data extracted from Han *et. al* 2014 [1] (article reports the same cohort as Han *et al*. 2017 [2]).

References

1. Han JH, Vasilevskis EE, Shintani A, Graves AJ, Schnelle JF, Dittus RS, et al. Impaired arousal at initial presentation predicts 6-month mortality: an analysis of 1084 acutely ill older patients. J Hosp Med. 2014;9:772-8.

2. Han JH, Brummel NE, Chandrasekhar R, Wilson JE, Liu X, Vasilevskis EE, et al. Exploring Delirium's Heterogeneity: Association Between Arousal Subtypes at Initial Presentation and 6-Month Mortality in Older Emergency Department Patients. Am J Geriatr Psychiatry. 2017;25:233-42.
